# Neutrophil-Airway Epithelial Interactions Result in Increased Epithelial Damage and Viral Clearance during Respiratory Syncytial Virus Infection

**DOI:** 10.1128/JVI.02161-19

**Published:** 2020-06-16

**Authors:** Yu Deng, Jenny A. Herbert, Elisabeth Robinson, Luo Ren, Rosalind L. Smyth, Claire M. Smith

**Affiliations:** a Infection, Immunity and Inflammation, UCL Great Ormond Street Institute of Child Health, London, United Kingdom; b Department of Respiratory Medical Centre, Chongqing Key Laboratory of Child Infection and Immunity, Children’s Hospital of Chongqing Medical University, China International Science and Technology Cooperation Base of Child Development and Critical Disorders, Ministry of Education Key Laboratory of Child Development and Disorders, Chongqing, China; University of North Carolina at Chapel Hill

**Keywords:** ALI, cilia, infection, inflammation, innate, neutrophil, respiratory

## Abstract

Respiratory syncytial virus (RSV) is a major cause of pediatric respiratory disease. Large numbers of neutrophils are recruited into the airways of children with severe RSV disease. It is not clear whether or how neutrophils enhance recovery from disease or contribute to its pathology. Using an *in vitro* model of the differentiated airway epithelium, we found that the addition of physiological concentrations of neutrophils to RSV-infected nasal cultures was associated with greater epithelial damage with lower ciliary activity, cilium loss, less tight junction expression (ZO-1), and more detachment of epithelial cells than is seen with RSV infection alone. This was also associated with a decrease in infectious virus and fewer RSV-positive cells in cultures after neutrophil exposure than in preexposure cultures. Epithelial damage in response to RSV infection was associated with neutrophil activation (within 1 h) and neutrophil degranulation, with significantly greater cellular expression of CD11b and myeloperoxidase and higher levels of neutrophil elastase and myeloperoxidase activity in apical surface media than in media with mock-infected airway epithelial cells (AECs). We also recovered more apoptotic neutrophils from RSV-infected cultures (>40%) than from mock-infected cultures (<5%) after 4 h. The results of this study could provide important insights into the role of neutrophils in host response in the airway.

**IMPORTANCE** This study shows that the RSV-infected human airway drives changes in the behavior of human neutrophils, including increasing activation markers and delaying apoptosis, that result in greater airway damage and viral clearance.

## Abstract (precis)

This study shows that the RSV-infected human airway drives changes in the behavior of human neutrophils, including increasing activation markers and delaying apoptosis, that result in greater airway damage and viral clearance.

## INTRODUCTION

Respiratory syncytial virus (RSV) is the major viral cause of pulmonary disease in young infants and the elderly and is responsible for annual epidemics that cause considerable morbidity and mortality worldwide (1, 2). A growing body of evidence suggests that the virus initiates infection by targeting human ciliated epithelium lining the nasopharynx (3–6). Recent advances in cell culture have allowed us to explore the early effects of RSV infection on ciliated human respiratory epithelium and helped elucidate the mechanisms by which RSV causes disease.

Severe RSV disease is characterized by profound neutrophilic inﬂammation in the lungs: up to 85% of bronchoalveolar lavage (BAL) cells from babies with bronchiolitis are neutrophils (7). These cells are thought to play an important role in host defense during respiratory virus infections, but they have also been implicated in lung tissue damage in a variety of conditions, including acute respiratory distress syndrome (ARDS), acute lung injury, cystic fibrosis (CF), and chronic obstructive pulmonary disease (COPD) (8–10). In these and other conditions, it may be that neutrophils recruited to the airways as part of host defense contribute to tissue damage and exacerbate disease. Currently, little is known about how neutrophils behave in the airways during RSV disease. Studies using animal models (11) (e.g., human RSV infection in mice, sheep, and cotton rats) lack sensitivity and do not fully recapitulate the disease in humans. For example, only 18% to 27% of BAL cells recovered from human RSV-infected calves, 3% to 14% from mice (12, 13), and <5% from cotton rats are neutrophils (14). In the human airway, infiltrating neutrophils directly interact with inflamed epithelial surfaces and have adapted mechanisms to respond to RSV-infected airway epithelial cells. However, few studies have investigated neutrophil function during this interaction. These functions include activation, degranulation, apoptosis (programmed cell death), and NETosis (cell death that is characterized by the discharge of decondensed chromatin and granular contents), which can affect the level of inflammation and tissue damage. The primary aims of this study were to evaluate the possible injury caused by neutrophils to RSV-infected ciliated cells in culture, to evaluate the early changes in neutrophil function during RSV respiratory infection, and to assess whether neutrophils had an antiviral effect.

## RESULTS

### Neutrophils enhance ciliated epithelial layer disruption and cilium loss.

**FIG 1:**
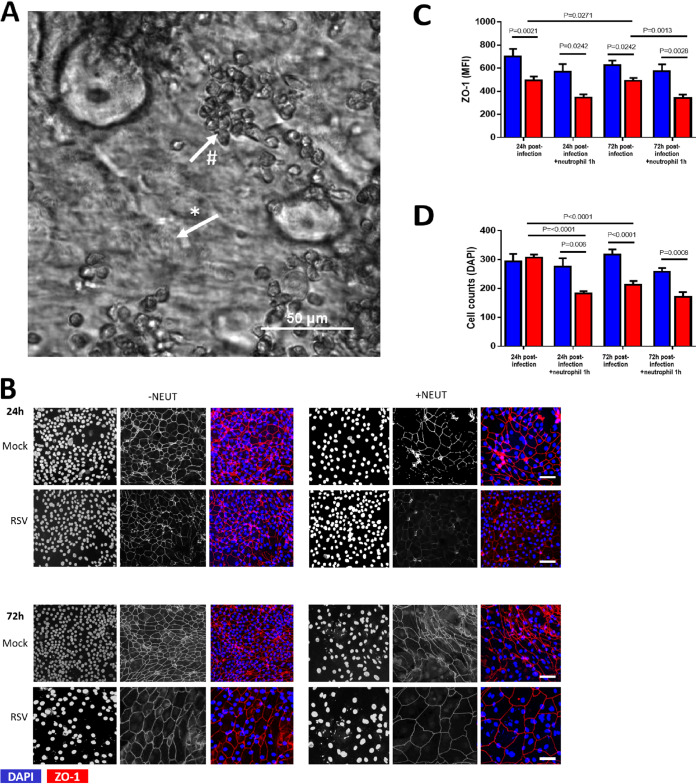
Epithelial damage caused by neutrophil exposure to RSV-infected ciliated epithelial cells. The effect of respiratory syncytial virus (RSV) and neutrophil exposure on the number of α-tubulin-positive cells. (A) Bright-field image of an RSV-infected culture after 4 h of coculture with neutrophils, showing ciliated epithelial cells (*) and neutrophil clusters (#). Scale bar shown. (B) Representative confocal images of RSV-infected human nasal ciliated epithelial cells with and without neutrophil exposure for 1 h. Cells were stained with antibodies against ZO-1 to detect tight junctions and with 4′,6-diamidino-2-phenylindole (DAPI) to detect epithelial nuclei. (C) Mean fluorescence intensity of ZO-1 for ciliated airway epithelial cells mock infected (blue bars) or infected with RSV (red bars) for 24 h or 72 h and after 1 h with neutrophils (*n* = 6 epithelial donors, 6 heterologous neutrophils). Bars represent the mean ± SEM. *P* values show a significant difference. (D) The number of epithelial cells attached to membrane inserts after neutrophil exposure. Epithelial cells were quantified by counting the DAPI-stained nuclei of >50 μm^2^ in area using ImageJ; the mean number (±SEM) of epithelial cells from all images is shown (*n* = 5 images per donor, 3 epithelial donors with heterologous neutrophils).

We found that neutrophils interacted with motile ciliated nasal airway epithelial cells (nAECs) almost immediately after introduction and gathered in clusters (Fig. 1A). After 1 h of incubation with ciliated nAECs infected with RSV for 24 h and 72 h, neutrophils were shown to decrease the epithelial expression of ZO-1 (a marker of epithelial cell tight junction proteins) compared with mock-infected cultures (*P* = 0.002). This reduction in ZO-1 expression was also significantly lower than in cultures of RSV-infected cells without neutrophils (Fig. 1B and C) at 72 h (*P* = 0.001), but not at 24 h postinfection (*P* = 0.07). We also found that the addition of neutrophils was associated with greater nAEC loss after 1 h, with significantly fewer RSV-infected nAEC nuclei remaining attached to the membrane insert at 24 h post-RSV infection than in cultures with the respective mock-infected control (*P* = 0.006) and RSV-infected nAECs without neutrophils (*P* < 0.0001) (Fig. 1D). ZO-1 staining and the numbers of epithelial cells were similar whether neutrophils were added to cultures infected for 24 or 72 h.

As RSV has been shown to target ciliated cells for infection, we were especially interested in how ciliary activity is altered during neutrophil interactions with RSV-infected epithelial cells. We found that ciliary beat frequency was unaffected by RSV infection or exposure over the entire study period (Table 1). However, RSV infection led to a higher proportion of dyskinetic cilia (Table 1). The mean dyskinesia index was significantly higher at 24 h post-RSV infection (25.71% ± 1.14%) and after 72 h of RSV infection (42.38% ± 2.44%) than mock-infected controls (8.44% ± 0.20% and 10.50% ± 0.35%, respectively) (*P* < 0.001).

**TABLE 1:** The ciliary beat frequency, dyskinesia index, and motility index of healthy nasal respiratory epithelial cells in pseudostratiﬁed air-liquid interface cultures infected with RSV A2 for 24 h or 72 h and then cocultured for 1 or 4 h with human neutrophils

Time of RSV infection (h)	Time of neutrophil coculture (h)	Mean beat frequency (Hz) of:		Mean dyskinesia index (%)^*a*^ of:		Mean motility index^*b*^ of:	
		Control	RSV infected	Control	RSV infected	Control	RSV infected
24	0	10.84 ± 0.28	11.64 ± 0.16	8.439 ± 0.20	25.71 ± 1.13*	63.51 ± 1.66	61.4 ± 1.67
	1	12.65 ± 1.00	12.56 ± 0.48	9.582 ± 0.28	25.77 ± 1.15*	68.37 ± 2.43	60.86 ± 1.82
	4	12.23 ± 1.07	12.08 ± 0.74	10.04 ± 0.84	26.91 ± 2.41*	65.1 ± 1.73	61.63 ± 2.35
72	0	12.76 ± 0.35	13.65 ± 0.28	10.50 ± 0.35	42.38 ± 2.44*	70.78 ± 1.97	57.6 ± 1.89*
	1	12.12 ± 1.59	11.29 ± 0.98	11.03 ± 0.45	49.45 ± 2.03*	66.94 ± 2.93	50.41 ± 2.77*#
	4	11.71 ± 1.30	10.87 ± 1.07	10.23 ± 0.59^*a*^	44.96 ± 2.62*	71.02 ± 2.36	65.4 ± 3.71

a Motility index, number of motile ciliated cells per sample area of ∼4.2 mm^2^.

b Dyskinesia index, percentage of abnormally beating cilia among all cilia examined. Data are expressed as mean ± SEM. Values highlighted in boldface were significantly different. *, *P* < 0.05 compared with mock. #, *P* < 0.05 compared to 0 h.

**FIG 2:**
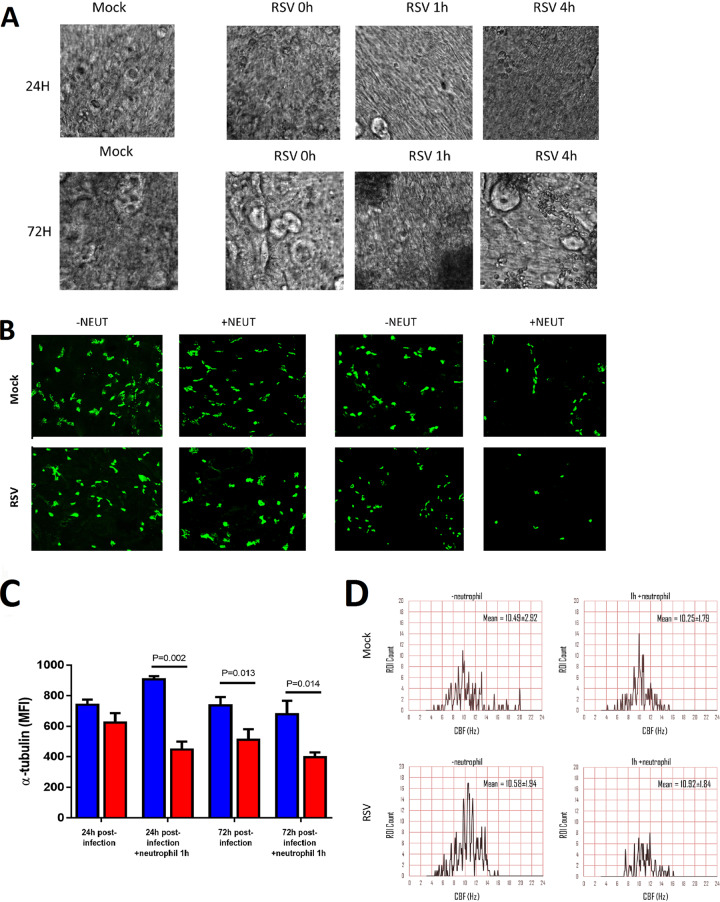
The number of ciliated epithelial cells decreases after neutrophil exposure. (A) Screenshots of slow-motion video microscopy files showing ciliated nasal epithelial cells following RSV infection and neutrophil exposure (see supplemental files 1 to 8). (B) Representative confocal images of RSV-infected human nasal ciliated epithelial cells infected for either 24 h (left panels) or 72 h (right panels) following neutrophil exposure for 1 h. Cells were stained with antibodies against acetylated tubulin to detect the ciliary axonemal microtubules (white). (C) The number of ciliated cells present on membrane inserts were quantified 24 h or 72 h postinfection and 1 h post-neutrophil exposure. The level of α-tubulin staining was used as a measure of cilia present (*n* = 5 images per donor, 3 epithelial donors with heterologous neutrophils). Bars represent the mean ± SEM for mock-infected (blue bars) or RSV-infected (red bars) cultures. (D) Histograms of the frequency distribution of ciliary beat frequency from 1,600 regions of interest taken from a representative field of view.

The addition of neutrophils to RSV-infected nAECs did not further enhance ciliary dyskinesia at any time point or infection condition tested (Table 1). Representative slow-motion videos showing ciliary beating are available as supplemental files 1 to 8 (screenshots are shown in Fig. 2A). We found no significant difference in mean fluorescence intensity (MFI) for α-tubulin staining (ciliary tubulin marker) under fluorescence microscopy examination at 24 h post-RSV infection compared to that for mock-infected cells or to that following 24 h of RSV infection with and without neutrophil exposure (Fig. 2B and C). However, we found that at 72 h post-RSV infection, the mean fluorescence intensity for α-tubulin was almost half that of the mock-infected group with neutrophils (*P* = 0.014) (Fig. 2C). This loss of α-tubulin staining correlated with a loss in the number of motile cilia observed by light microscopy (referred to as the mean motility index) at 72 h post-RSV infection (57.6% ± 1.89%), which was lower than that of the mock-infected controls (70.8 %± 1.97%) (*P* < 0.05). Exposure to neutrophils for 1 h lowered the mean motility index of ciliated cells infected with RSV for 72 h (50.41 ± 2.77%) compared with the preneutrophil time point (*P* < 0.05) (Table 1). As is shown in Fig. 2D, the distribution of ciliary beat frequency of the same field of view before and after the addition of neutrophils produced a similar mean ciliary beat frequency (CBF) of around 10.2 to 10.9 Hz, but fewer areas (region of interest [ROI]) from the RSV-infected ciliated nAECs (bottom right panel of Fig. 2D) show active beating cilia (defined as >3 Hz) when cocultured with neutrophils.

### Incubation with neutrophils reduces the amount of infectious virus in the culture.

**FIG 3:**
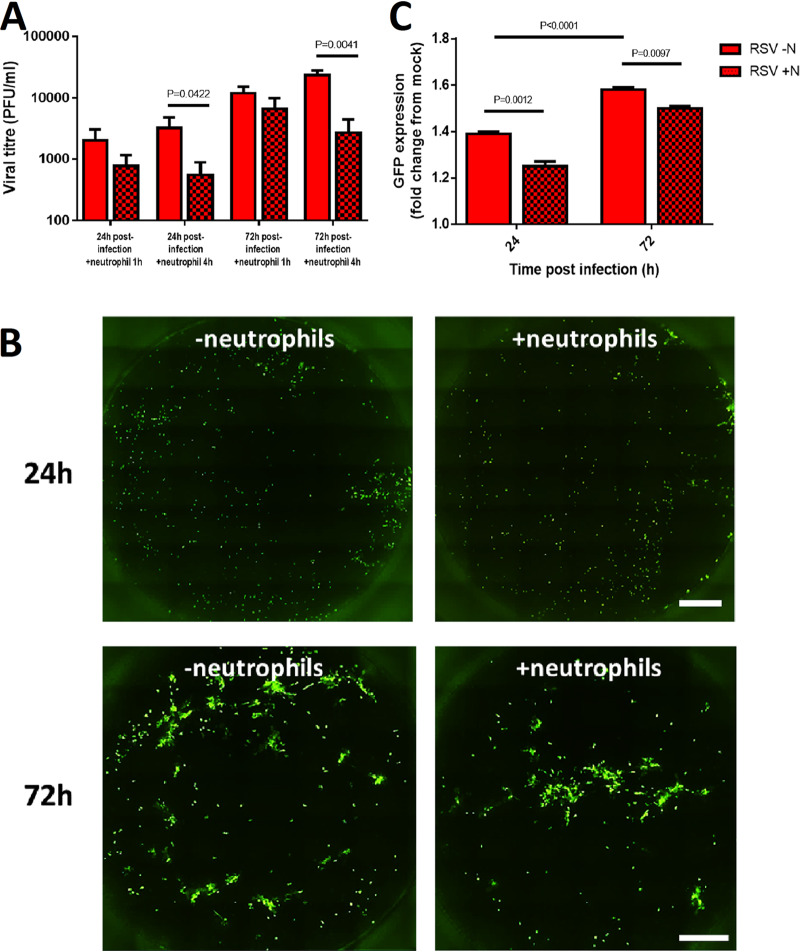
The amount of infectious RSV is decreased in infected ciliated epithelial cells exposed to neutrophils. (A) The amount of infectious virus present in whole wells following neutrophil coculture as measured by plaque forming units per milliliter (PFU/ml). (B) Whole-well scan of a representative membrane insert infected with RSV for 24 h or 72 h cells before (left) and 1 h after (right) neutrophil exposure. Each white spot indicates an RSV-infected epithelial cell. (C) Mean fluorescence intensity of GFP-RSV-infected AECs for 24 h or 72 h cells after 1 h without (clear bars) and with (spotted bars) neutrophils. Bars represent the mean ± SEM (*n* = 6 epithelial donors with heterologous neutrophils. *P* values show a significant difference between the matched pre- and post-neutrophil exposure time points.

Four hours after neutrophil exposure, we detected significantly smaller amounts of infectious virus, with an RSV titer of 2.4 × 10^4^ PFU/ml recovered from cultures infected with RSV for 72 h without neutrophils, compared with 2.8 ×10^3^ PFU/ml for cultures infected with RSV for 72 h with neutrophils (*P* < 0.05) (Fig. 3A). We assessed the numbers of RSV-infected nAECs by measuring the mean fluorescence intensity of GFP, a marker of active cytoplasmic RSV replication. Using fluorescence microscopy (Fig. 3B), we found that GFP expression was significantly lower 1 h after exposure to neutrophils than for cells not cocultured with neutrophils at both 24 h and 72 h postinfection (*P* < 0.05) (Fig. 3B and C).

### Incubation with RSV-infected ciliated epithelial cells increases neutrophil apoptosis.

**FIG 4:**
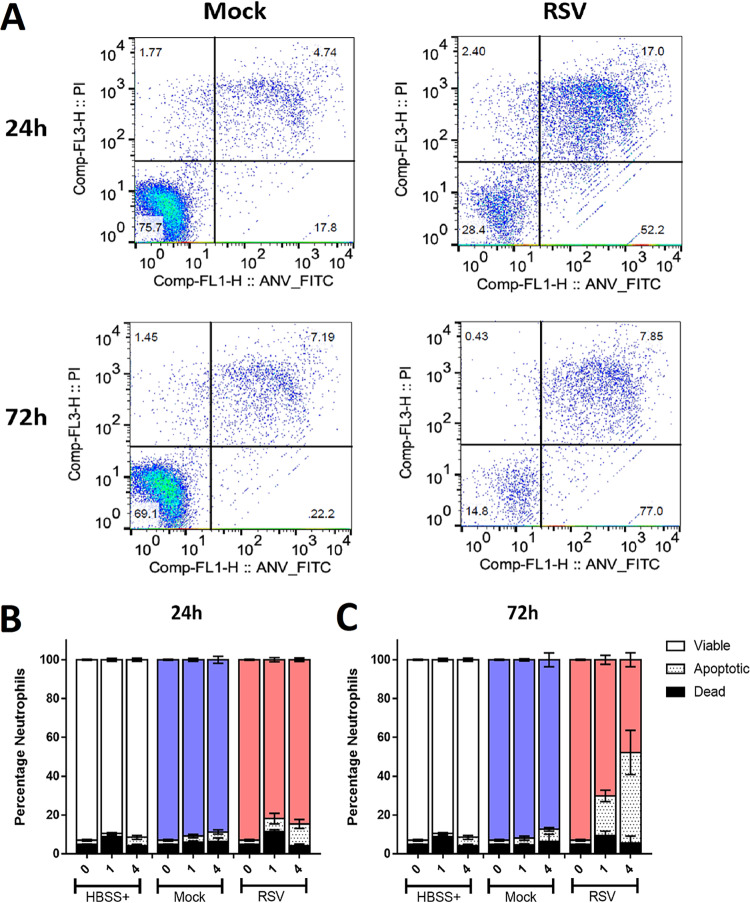
Neutrophil viability and apoptosis following exposure to RSV-infected ciliated epithelial cells. (A) Viability was calculated by the exclusion of propridium iodide (PI^lo^); apoptosis was determined by expression of annexinV (FITC^hi^) and determined by flow cytometry. Neutrophils were first identified using a CD11b (APC)-positive gate. Using this proportion of the population with fluorescence intensity of PI and/or FITC, fluorescence was calculated. Q1, PI^hi^ANV^lo^; Q2, PI^hi^ANV^hi^ (Q1 + Q2 = dead neutrophil); Q3, PI^lo^ANV^lo^ (viable neutrophils); Q4, PI^lo^ANV^hi^ (apoptotic neutrophils). (B and C) Bars show means ± SEM (*n* = 4 to 7 epithelial donors with heterologous neutrophils) for HBSS (white bars), mock-infected (blue bars), or RSV-infected (red bars) cultures 24 h (B) or 72 h (C) postinfection. Statistical significance is shown.

Using flow cytometry (Fig. 4A), we showed that at 24 h post RSV-infection, there was no difference in the percentage of apoptotic (PI^lo^ AnnexinV^hi^) neutrophils recovered from RSV-infected nAECs (11.1% ± 5.1%) compared with 4.8% ± 2.7% in the mock-infected cocultures (Fig. 4B). However, at 72 h postinfection, we detected significantly more apoptotic neutrophils after 4 h of exposure to RSV-infected nAECs with 46.7% ± 11.4%, compared with 6.2% ± 0.9% in the mock-infected cocultures (*P* < 0.0001) (Fig. 4B). Apoptosis appeared to be the dominant form of cell death, as we did not detect an increase in dead (PI^hi^ AnnexinV^lo^) neutrophils at any time point or test condition. After 4 h of exposure to epithelial cells infected with RSV for 72 h, we detected 6.43% ± 7.2% dead neutrophils compared with 5.5% ± 7.3% in the mock control (*P* > 0.99) (Fig. 4B).

### Incubation with RSV-infected ciliated epithelial cells increases neutrophil expression of CD11B and MPO and augments degranulation.

**FIG 5:**
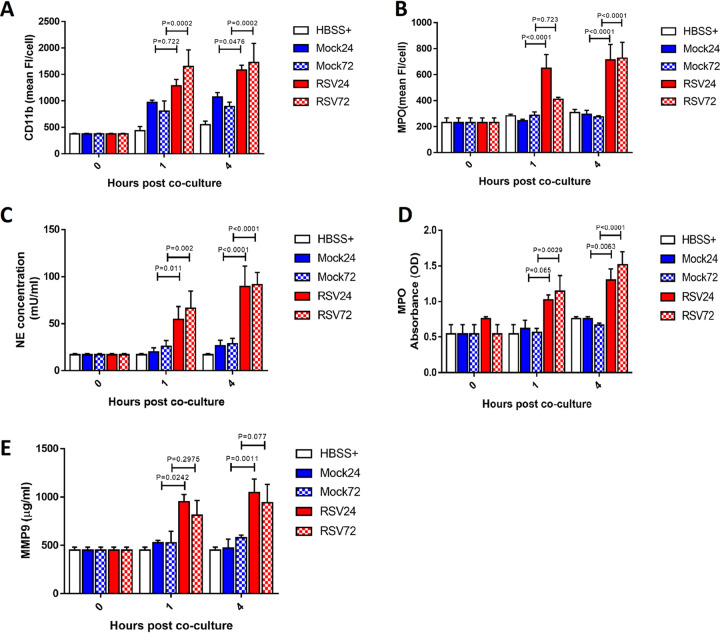
Neutrophil activation and release of neutrophil-derived products and neutrophil activation after exposure to RSV-infected ciliated epithelial cells. Activation marker expression was calculated by staining for cell surface expression of CD11b (PE^hi^) or MPO (allophyocyanin [APC^+^]) and determined by flow cytometry. Neutrophils were identified using a PE-positive gate. Using this population, the geometric mean fluorescence intensity of PE or APC fluorescence was calculated. (A) Neutrophils exposed to RSV (red bars)-infected epithelial cells infected for 24 h (plain bars) or 72 h (checkered bars) show increased cell surface expression of CD11b compared to those exposed to mock (blue bars)-infected epithelial cells. (B) Neutrophils exposed to RSV (red bars)-infected epithelial cells infected for 24 h (plain bars) or 72 h (checkered bars) show increased cell surface expression of MPO compared to those exposed to mock (blue bars)-infected epithelial cells. Bars show means ± SEM (*n* = 5 to 8 epithelial donors with heterologous neutrophils). Statistical significance is shown. Apical surface medium concentrations of neutrophil elastase (NE) (C), MPO (D), and MMP-9 (E) were measured in the apical supernatant by ELISA collected following neutrophil exposure to mock- or RSV-infected (24 h [plain bars] or 72 h [checkered bars]) ciliated AECs after 1 or 4 h. For all graphs, bars represent the mean ± SEM (*n* = 5 epithelial donors with heterologous neutrophils for HBSS+ (white), mock-infected (blue bars), or RSV-infected (red bars) cultures. A statistical comparison between all groups was performed using a paired *t* test. Statistical significance is shown.

Incubation with ciliated epithelial cells RSV infected for 24 h led to significantly greater expression of CD11B on neutrophils after 4 h, but not after 1 h, than on neutrophils that were incubated with mock-infected ciliated epithelial cells, with a mean (±SEM) fluorescence intensity of 1.3 × 10^3^ ± 1.21 × 10^2^ compared to 9.7 × 10^2^ ± 3.9 × 10^1^ after 4 h (*P* = 0.048) (Fig. 5A). At 72 h postinfection, incubation with ciliated epithelial cells infected with RSV led to significantly greater expression of CD11B on neutrophils after 1 h and 4 h than on neutrophils that were incubated with mock-infected ciliated epithelial cells, with MFIs of 1.7 × 10^3^ ± 3.6 × 10^2^ and 8.9 × 10^2^ ± 8.2 × 10^1^, respectively, after 4 h (*P* = 0.0002) (Fig. 5A). Incubation with epithelial cells infected for 24 h showed significantly greater expression of myeloperoxidase (MPO) on neutrophils after 1 h and 4 h of incubation than on neutrophils that were incubated with mock-infected ciliated epithelial cells, with a mean (±SEM) fluorescence intensity of 6.5 × 10^2^ ± 1.1 × 10^2^ compared with 2.5 × 10^2^ ± 1.2 × 10^1^ after 24 h of infection (*P* < 0.0001) (Fig. 5B), with similar findings at 1 h (*P* = 0.722) and 4 h (*P* < 0.0001) after 72 h of RSV infection.

Neutrophils incubated with RSV-infected ciliated epithelial cells released more active neutrophil elastase (NE) at 24 h and 72 h post-RSV infection (*P* < 0.05) (Fig. 5C). We found that the concentration of NE in apical surface media was significantly greater after 1 h of neutrophil exposure to RSV-infected AECs at both 24 h (*P* = 0.01) and 72 h (*P* = 0.002) postinfection, with a mean ± SEM of 54.5 ± 13.5 mU/ml compared with 19.8 ± 4.4 mU/ml in the mock-infected cultures at 24 h postinfection. To determine whether NE correlated with augmented azurophil granule release or was specific to NE alone, MPO activity was also assessed. Here, we found significantly more MPO activity (*P* = 0.003) after exposure of neutrophils for 4 h to epithelial cells infected with RSV for 24 h (*P* = 0.006) or 72 h (*P* < 0.0001) (Fig. 5D), with a mean ± SEM of 1.0 ± 0.0 optical density (OD) at 24 h, than 0.6 ± 0.1 OD in the mock-infected cultures. This was also significantly different after 1 h at 72 h (*P* < 0.003) but not 24 h (*P* = 0.065) post-RSV infection.

RSV infection was also associated with a greater release of active MMP-9 from neutrophils (Fig. 5E) after 1 h and 4 h (*P* = 0.001) in cultures infected with RSV for 24 h, from 529.4 ± .21.9 μg/ml in the mock to 949.3 ± 76.9 μg/ml in RSV-infected epithelial cells at 1 h (*P* = 0.02). There was no significant difference in MMP-9 concentrations between RSV- and mock-infected cocultures at 72 h postinfection at either 1 h (*P* = 0.30) or 4 h (*P* = 0.07).

## DISCUSSION

We have shown that when RSV infected human primary nasal airway epithelial cells are exposed to neutrophils at physiological concentrations, there is increased epithelial layer disruption, ciliary loss, and less infectious virus in these cultures. This suggests that neutrophils are helping to eliminate virus-infected cells and reduce viral spread. The airway epithelial damage that we observed, consistent with previous studies (15, 16), may be a necessary consequence of this antiviral effect. We found that RSV infection without neutrophils did not reduce CBF but did increase the number of cilia that presented with an abnormal beat pattern as early as 24 h postinfection, which is similar to our previous findings (3, 5). Interestingly, the addition of neutrophils did not further increase ciliary dyskinesia.

The reduction in the number of RSV-infected epithelial cells following neutrophil exposure may result from neutrophil degranulation. Neutrophils are known to mediate direct antimicrobial effects and neutralize several influenza A, RSV, and vaccinia virus strains through effector mechanisms, including degranulation (17–19). We have shown that neutrophils exposed to RSV-infected human primary airway epithelial cells have greater expression of the activation markers CD11B and MPO and release greater amounts of NE, MPO, and MMP-9. It is recognized that the inflammatory processes in the airways of infants with RSV bronchiolitis are dominated by an intense neutrophil influx (7, 20) and that neutrophil products, such as MPO and NE, are released into the airway lumen (21). Indeed, the degree of neutrophilic inflammation correlates with disease severity in patients with RSV-induced bronchiolitis (22). Our study has shown that RSV-infected epithelial cells increased NE and MPO and gelatinase (MMP-9) granule concentrations, at the same time reducing neutrophil membrane integrity. This appears to be relevant to the pathophysiology of viral respiratory infections (23, 24). Likewise, both NE and MMP-9, which may be necessary for clearance of bacteria, are linked to airway damage and the progression of cystic fibrosis (25). Our results are consistent with these clinical findings, suggesting that the cytotoxicity of neutrophil antimicrobial proteases may be important in viral clearance but may also potentiate RSV-induced lung injury.

**FIG 6:**
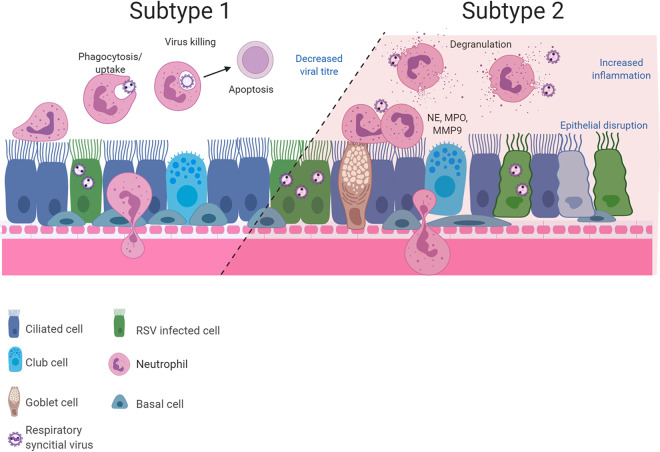
Diagram showing the two possible neutrophil subsets that may be present during RSV infection of ciliated airway epithelial cells. This hypothesis may explain how simultaneous infection resolution and pathogenesis occur in our model, with results showing increased neutrophil activation, clearance of RSV, and neutrophil apoptosis (subtype 1). Our model also demonstrated neutrophil degranulation and release of proteases which are known to damage the epithelium (subtype 2). Drawings were created with BioRender.

A surprising finding was that at 72 h, but not at 24 h, post-RSV infection, neutrophil exposure led to an increase in the numbers of apoptotic neutrophils. This finding is consistent with a clinical study that found neutrophil apoptosis was accelerated in nasopharyngeal aspirates and peripheral blood of infants with RSV bronchiolitis (26). Neutrophil apoptosis is thought to be associated with reduced levels of degranulation and other proinflammatory capacities (27). We found that RSV-infected epithelial cells increased neutrophil degranulation in regard to increased neutrophil elastase (NE) and myeloperoxidase (MPO) activity in apical surface media at the same time as we detected the increased neutrophil apoptosis. These data suggest that within this model, there may be at least two subsets of neutrophils that respond differently to RSV-infected airway epithelial cells. It is possible that this balance in neutrophil function could be a predictor of disease severity (see Fig. 6).

One limitation to our study was the number of neutrophils used. The exact ratio of interacting neutrophils and airway epithelial cells in the lungs of children with RSV bronchiolitis is unknown. We used the equivalent concentration of 5 × 10^6^/ml neutrophils, which is the upper limit of the number of neutrophils recovered from BAL specimens of infants with RSV bronchiolitis (1.78 ± 3.3 × 10^6^/ml) that was reported previously by McNamara and colleagues (7). This suggest that our data may indicate airway-immune cell interactions that occur in the lungs of infants with large neutrophil infiltrate or severe RSV bronchiolitis. Another limitation of our study was that we exposed epithelial cells to naive neutrophils directly isolated from peripheral blood. In the lungs, neutrophils are recruited to migrate from the basal subepithelial space across the vasculature and epithelium to the airways following RSV infection (28). In this context, the changes that we observed are all the more striking.

In conclusion, this study has revealed that neutrophils exposed to ciliated epithelial cell cultures infected with RSV have increased degranulation, deploying harmful proteins and proteases to the apical surface media and increasing the capacity for tissue injury. We have shown that neutrophils contribute to RSV-associated ciliary loss combined with epithelial damage, which is likely to result in reduce mucociliary clearance. These effects may contribute to viral clearance and provide important insights into the role of neutrophils in host responses in the airway.

## MATERIALS AND METHODS

### Subjects and ethical considerations.

Human nasal epithelial cells and/or peripheral blood samples were obtained from different (heterologous) adult healthy control donors who had no history of nasal or respiratory disease. Epithelial cells were collected at least 3 months prior to neutrophil collection. None of the subjects were taking medications or had reported a symptomatic respiratory infection in the preceding 6 weeks. All samples were obtained with the individual’s permission and with ethical approval by the UCL Research Ethics Committee (ref 4235/002).

### Viral infection of primary epithelial cell cultures.

**FIG 7:**
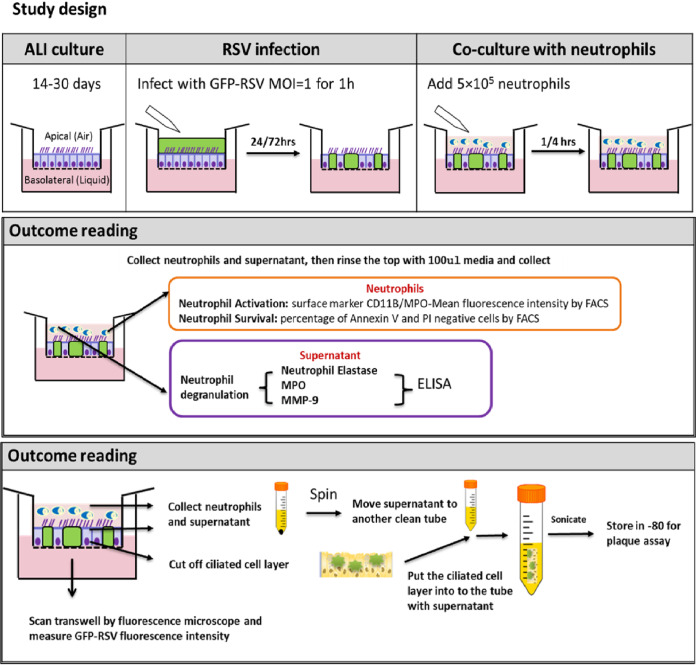
Schematic diagram of our study design. Diagram of air-liquid interface (ALI) cell culture, airway epithelial cell infection with GFP-RSV, and neutrophil coculture. Transwell inserts containing ciliated differentiated airway epithelial cells were infected with GFP RSV or mock infected for 1 h before the inoculum was removed. Transwell inserts were maintained in media and infection was allowed to progress for 24 or 72 h. Ultrapure (>99.9%) neutrophils (5 × 10^5^) were then added to the apical side of the Transwell inserts and cocultured for 1 or 4 h. After coculture, the apical neutrophils were collected and downstream analysis was performed.

Primary human nasal airway epithelial cells (nAECs) were grown to a ciliated phenotype on 0.4-μm pore Transwell inserts (Corning) at air-liquid interface (ALI) as described previously (3). Recombinant GFP-tagged RSV A2 strain was kindly provided by Fix et al. (29) and propagated using HEp-2 cells (multiplicity of infection [MOI], 0.1) for 3 to 5 days in Opti-MEM. Virus was purified as described previously (30), collected in bronchial epithelial basal medium (BEBM; Life Technologies), and frozen at –80°C. Aliquots of RSV were thawed immediately prior to use. For ALI culture infection, the apical surface of the ALI cultures was rinsed with medium (BEBM), and 200-μl viral inoculum (MOI, 1) in BEBM was applied to the apical surface for 1 h at 37°C and then removed (see Fig. 7). Mock wells received BEBM alone. All cells were fed basolaterally with fresh ALI medium prior to infection. The infection continued for 24 h and 72 h.

### Neutrophil isolation and purification.

Neutrophils were isolated from peripheral venous blood using a Percol density gradient, as described previously (31), and were purified using an EasySep human neutrophil enrichment kit (Stemcell Technologies) per the manufacturer’s instructions (32). Neutrophils were resuspended in Hanks balanced salt solution (HBSS−) (Life Technologies), and fluorescence-activated cell sorter (FACS) analysis was performed using anti-human CD49d-APC (BioLegend, 304307) and anti-human CD66a/c/e Alexa Fluor-488 (BioLegend, 342306) antibodies to confirm purity. Neutrophils were counted using a hemocytometer.

### Neutrophil airway epithelial cell coculture.

Neutrophils (5 × 10^5^ in 100 μl HBSS+) were added to the top (apical) chamber of Transwell inserts containing either RSV- or mock-infected ciliated nAECs and incubated at 37°C + 5% CO_2_. After 1 h or 4 h of incubation, neutrophils and apical surface media from the top chamber of Transwell were collected and each membrane was washed once with 0.1 ml HBSS+. The apical surface media and washing medium were pooled, spun, and stored at –80°C. The cell pellet was resuspended in 500-μl FACS buffer (phosphate-buffered saline [PBS] [Ca2+Mg2+ free], 0.5% bovine serum albumin [BSA], and 2.5 mM EDTA) for further analysis.

### AnnexinV/PI apoptosis assay.

The annexin V apoptosis detection kit (Miltenyl Biotec) was used to carry out this assay. Neutrophils collected from the cell pellet (described above) were resuspended in 50-μl FACS buffer plus 1:250 dilution APC anti-human CD11B conjugate (Insight Biotechnology) and incubated at 4°C for 20 minutes in the dark. Cells were washed once in FACS buffer, resuspended in 50-μl annexin binding buffer with 0.3 μl of annexin V for 15 minutes in the dark at room temperature. They were then flash stained with propidium iodide (PI), 10 μl in 900 μl of annexin binding buffer, and analyzed on a FACSCalibur flow cytometer. Unstained and annexin V or PI single-stained controls confirmed no cross-reactivity. At least 10,000 events were collected. Neutrophils were first identified as being CD11B positive (APC+). Early apoptosis was identified by annexin V positivity and cell death by PI staining.

### Neutrophil CD11B and MPO expression.

Neutrophil activation was determined by measuring the cell surface protein expression levels of CD11B and MPO. All neutrophils were centrifuged at 1,400 rpm for 5 mins and washed once in 500-μl FACS buffer (PBS [Ca2+Mg2+ free], 0.5% BSA, and 2.5 mM EDTA). The cell pellet was then resuspended in 50 μl (1/50 dilution in FACS buffer) of TruStain FcX blocker antibody (Biolegend), incubated at 4°C for 10 minutes, and then washed in FACS buffer as above. Cells were resuspended in 50-μl FACS buffer plus 1/250 dilution PE anti-human CD11B conjugate (50-0118-T100, Insight Biotechnology) and 1/50 dilution anti-MPO-APC human antibody (130-107-177, clone:REA491; Miltenyl Biotec) and incubated at 4°C for 20 minutes in the dark. Unstained and single-antibody controls confirmed no cross-reactivity. Cells were washed once in FACS buffer, resuspended in 1% (wt/vol) paraformaldehyde (PFA), and stored at 4°C. Directly prior to being run, samples were centrifuged (1,400 rpm) and resuspended in FACS buffer.

Samples were analyzed using a Beckton Dickenson LSR II flow cytometer and FlowJo v10.0 FACS analysis software. Neutrophils were first identified as being CD11B positive (PE+). Using this population, the mean fluorescence intensity for PE and APC was calculated.

### Determination of neutrophil degranulation.

Degranulation was assessed by measuring neutrophil elastase (NE), myeloperoxidase (MPO), and matrix metalloproteinase-9 (MMP-9) in the apical surface media. The amount of NE or MPO in apical surface media was measured using commercial activity assay kits (Cayman, USA). MMP-9 release was measured using a commercial ELISA kit (Biolegend, USA). All protocols were conducted according to the manufacturers’ instructions.

### CBF and beat pattern.

Beating cilia were observed via an inverted microscope system (Nikon TiE; Nikon, UK) equipped with an incubation chamber (37°C and 5% CO_2_) as previously described (3). To determine ciliary beat frequency (CBF), videos were recorded using a 20× objective using a CMOS digital video camera (Hamamatsu) at a rate of 198 frames per second and image size of 1,024 by 1,024 pixels. CBF (Hz) was calculated using ciliaFA software (33). The number of motile ciliated cells in each sample area was counted (motility index). The dyskinesia index was calculated as the percentage of dyskinetic ciliated cells (those that displayed uncoordinated motile cilia or those that beat with a stiff, flickering, or twitching motion) relative to the total number of motile ciliated cells.

### Immunofluorescence microscopy.

Following fixation, cells were washed three times with PBS and treated with PBS containing 0.1% Triton X-100 for 10 min to permeabilize the cells. Cells were incubated with 5% fetal calf serum (FCS) in PBS for 0.5 h at room temperature to block nonspecific interactions and washed again three times with PBS. All subsequent antibody incubations were carried out in 5% FCS in PBS + 0.1% Triton X-100. Reagents used in this study were rabbit anti-ZO-1 polyclonal antibody (1:200; catalog number sc-5562; Santa Cruz Biotechnology) and mouse anti-acetylated α-tubulin monoclonal antibody (6-11B-1; 1 μg/ml; Sigma). Primary antibody incubations were carried out in a humidified chamber overnight at 4°C, followed by three washes with PBS. The detection of primary antibodies was carried out for 1 h using the following reagents: fluorescein fluorescein isothiocyanate (FITC; catalog number F2012; Sigma) conjugated rabbit anti-mouse (1:64) or Alexa Fluor 594-conjugated rabbit anti-donkey antibody (1:250; Invitrogen, Paisley, UK). All secondary antibodies were tested and found to be negative for cross-reactivity against human epithelial cells. Following three washes in PBS, DNA was stained with Hoechst 33258. After a final wash in distilled water, the insert was cut from the support and mounted under coverslips in 80% (vol/vol) glycerol and 3% (wt/vol) n-propylgallate (in PBS) mounting medium. Images were captured with a confocal laser microscope (Zeiss Observer Z.1) using a 40× water immersion objective. The pinhole was set at 1 airy unit (AU). For z-stack images, the slice thickness was 1 μm.

### Statistical analysis.

Statistical analysis was performed using GraphPad Prism 5 (GraphPad, San Diego, CA, USA). Differences between the mock- and RSV-infected group were analyzed using paired *t* tests or a paired two-way analysis of variance (ANOVA) for multiple comparisons with a Bonferroni correction (GraphPad Prism v5.0).

## Supplementary Material

Supplemental file 1

Supplemental file 2

Supplemental file 3

Supplemental file 4

Supplemental file 5

Supplemental file 6

Supplemental file 7

Supplemental file 8

Supplemental file 9
