# Supplementary material for: Neutrophil-Airway Epithelial Interactions Result in Increased Epithelial Damage and Viral Clearance during Respiratory Syncytial Virus Infection
Source: J Virol. 2020 Jun 16;94(13):e02161-19. doi: 10.1128/JVI.02161-19 (PMC7307165; doi:10.1128/JVI.02161-19)
Supplement: Supplemental file 9 [file JVI.02161-19-s0009.docx]

**Supplementary video legends**

Mock 24h (0h) : differentiated epithelial cells mock infected for 24h prior to addition of neutrophils

RSV 24h (0h) : differentiated epithelial cells RSV infected for 24h prior to addition of neutrophils

RSV 24h (1h+neuts) differentiated epithelial cells RSV infected for 24h and exposed to neutrophils for 1h

RSV 24h (4h+neuts) differentiated epithelial cells RSV infected for 24h and exposed to neutrophils for 4h

Mock 72h (0h) : differentiated epithelial cells mock infected for 72h prior to addition of neutrophils

RSV 72h (0h) : differentiated epithelial cells RSV infected for 72h prior to addition of neutrophils

RSV 72h (1h+neuts) differentiated epithelial cells RSV infected for 72h and exposed to neutrophils for 1h

RSV 72h (4h+neuts) differentiated epithelial cells RSV infected for 72h and exposed to neutrophils for 4h
